# Prototyping Mobile Storytelling Applications for People with Aphasia

**DOI:** 10.3390/s22010014

**Published:** 2021-12-21

**Authors:** Krzysztof Szklanny, Marcin Wichrowski, Alicja Wieczorkowska

**Affiliations:** Multimedia Department, Polish-Japanese Academy of Information Technology, 02-008 Warsaw, Poland; kszklanny@pjwstk.edu.pl

**Keywords:** aphasia, assistive technology, storytelling, user-centered design, graphical user interface, usability tests, mobile devices, wearable devices, smart glass, Google Glass

## Abstract

Aphasia is a partial or total loss of the ability to articulate ideas or comprehend spoken language, resulting from brain damage, in a person whose language skills were previously normal. Our goal was to find out how a storytelling app can help people with aphasia to communicate and share daily experiences. For this purpose, the Aphasia Create app was created for tablets, along with Aphastory for the Google Glass device. These applications facilitate social participation and enhance quality of life by using visual storytelling forms composed of photos, drawings, icons, etc., that can be saved and shared. We performed usability tests (supervised by a neuropsychologist) on six participants with aphasia who were able to communicate. Our work contributes (1) evidence that the functions implemented in the Aphasia Create tablet app suit the needs of target users, but older people are often not familiar with tactile devices, (2) reports that the Google Glass device may be problematic for persons with right-hand paresis, and (3) a characterization of the design guidelines for apps for aphasics. Both applications can be used to work with people with aphasia, and can be further developed. Aphasic centers, in which the apps were presented, expressed interest in using them to work with patients. The Aphasia Create app won the Enactus Poland National Competition in 2015.

## 1. Introduction

Aphasia can be caused by brain damage of various origins—e.g., stroke, brain tumor, or head trauma—located in the brain area associated with language processing. Generally, there are three types of aphasia, depending on the area and degree of brain damage: Expressive aphasia (Broca’s aphasia) is an impairment of the production of language; in this case, speech is impaired [1,2], and sentence construction is very poor—sometimes limited to only a few words or a single word. Receptive aphasia (Wernicke’s aphasia) negatively affects the comprehension of language—individuals do not understand spoken or read words. The combination of both cases results in global aphasia, wherein both the production and understanding of language become impossible [3,4]. An additional problem faced by people with aphasia is difficulty in remembering the chronology of events and the times when things happened. These problems significantly degrade the quality of the individuals’ lives, especially regarding interaction involving interpersonal communication [5]. Avoidance of social contact and the feeling of being rejected by part of society hinder their rehabilitation, and make it difficult to return to normal life.

To prevent such situations, various analogue forms are used to help people with aphasia to communicate, such as symbol books, photo albums, communication boards, etc. Along with the development of technology, digital augmentative and alternative communication (AAC) devices were introduced, helpful in daily basic use and during rehabilitation [6,7]. Unfortunately, most of these devices do not support creating, saving, and sharing stories from everyday life, which is crucial to maintaining familial and social bonds. The mainstream mobile applications with such functionalities are not tailored to the needs of aphasia patients.

Our goal was to prepare storytelling applications to be used by patients with Broca’s aphasia (also known as expressive aphasia) [1] after or in the course of rehabilitation, in order to improve their quality of life. Aphasics usually cannot speak nor write, so they use gestures, drawing or pointing to objects (or to themselves), symbols and photos, etc., in order to communicate their basic needs [8]. Our applications may replace these means of communication, and they may also be used at a more advanced state of rehabilitation to support more sophisticated communication, including storytelling. 

Moreover, we were interested in assessing the user experience (UX) aspects of the proposed solutions [9]—especially the designed user interfaces—and how they were perceived in the first contact with the applications.

### Storytelling Applications for Mobile Devices

The popularity of smartphones and tablets has changed the way digital technologies are used in the medical sector. Digital devices are small, portable, fast to operate, and reliable [10]; they are also becoming quite common among older people, and their versatility and connection to the Internet opens the way to provide a whole new quality of help for people with aphasia. Mobile technology has resulted in the emergence of new methods of interaction and possibilities. Improving access to mobile computing technology for people with aphasia has the potential to enhance both social participation and the management of aphasia. The key features of mobile devices for non-rehabilitative purposes and the potential role of smartphones as a cost-effective, time-efficient, and context-sensitive health management tool have become the focus of research [11].

Observations taken from [8] indicate that people with aphasia need to be listened to and understood, just like everybody else; they want to share their anecdotes, observations, ideas, and stories from everyday life; otherwise, there is a high risk of isolation and developing a passive role in social interactions, which can lead to stress and depression. Since many of these persons remain alone at home, they require easy-to-use applications that allow for quick communication over a distance. Aphasics can be reluctant to use solutions that would emphasize their disability, especially in social relations. The possibility of indirect communication (e.g., in the form of writing e-mails or creating drawings), for which they can take time and prepare properly, is received more positively.

The storytelling approach has the potential to activate people with aphasia, because most interpersonal contacts are based on conversations and sharing stories. The use of photographic images in storytelling as a basis for developing communication in patients with aphasia significantly improves the participants’ psychological wellbeing and recovery [12,13,14]. Applications offering this kind of interaction may be a potential solution to the problems from which people with expressive aphasia suffer. Storytelling applications most often allow for the building of visual stories based on photos or videos taken by the user, which can additionally be described with text, symbols, etc., and then shared with others [15]. Sometimes, face-to-face communication is also supported [16]. These solutions, apart from stimulating a person with aphasia to establish social contacts and communication, also facilitate the remembering and organization of past events in time, and support narration. Therefore, the storytelling approach has a significant impact on reducing the sense of isolation and the passive attitudes of patients toward their aphasia [15,17,18].

Mobile stores offer a variety of storytelling apps. A good example was the award-winning Storehouse; unfortunately, Storehouse services were shut down on 15 July 2016 [19]. Another application—Day One [20]—is an advanced personal and open-ended digital journaling solution. The Five Minute Journal App [21] encourages users to share observations by answering questions such as “I am grateful for…”, “What will I do to make today great?”, etc. Sometimes applications of this type are combined with mood tracking, e.g., the Reflectly app [22]. Unfortunately, despite the wide range of applications, they are not dedicated to people with aphasia; their main goal is to create diaries presented in an aesthetic form, rather than supporting storytelling for communication. In addition, their operation may exceed the capabilities of older people less experienced in using technology (the screens of these applications consist of a large number of complex screens, too many unnecessary functions, small icons, etc.). 

An interesting perspective, apart from tablets and smartphones, is given by even more personal devices, such as smart glasses. Glossaic—an application and social network service for Google Glass [23]—allows users to capture, describe, and share their favorite pictures and videos. Only Glass users can post pictures and videos in Glossaic, but anyone else can comment, rate, and like them. Memoirs [24] is another application for the Google Glass device that allows for documenting everyday memories in the form of a diary, creating and saving comments and photos, and downloading them from the website. To the best of our knowledge, no Google Glass storytelling apps have been developed for persons with aphasia, except for the app described below.

## 2. Materials and Methods

We designed and implemented two apps—Aphasia Create for mobile devices (to be used on tablets) and Aphastory for Google Glass—to support communication of persons suffering from expressive aphasia. 

### 2.1. Aphasia Create Mobile Application

The idea for the Aphasia Create app was born during the internship of one of the authors, Marcin Wichrowski, at Eindhoven University of Technology, with the cooperation of Professor Jean-Bernard Martens. The main source of inspiration was the Snap application prototype designed by Jamie Maria Schouren. The idea of this project was to communicate using a graphical mood board application, where users can share thoughts, experiences, emotions, and feelings.

**Figure 1:**
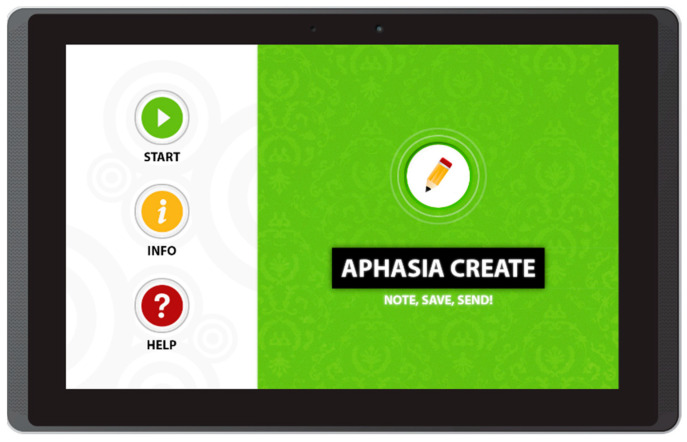
Main menu in Aphasia Create (screenshot from an Android tablet).

The Aphasia Create app also follows this approach, and extends it with new functionalities. The application has a very simple main menu with three buttons: Start, Info, and Help (Figure 1); it was implemented using Adobe AIR for Android- and IOS-operated mobile devices [25]. As we can see in Figure 1, the interface is very simple and the icons are relatively large.

**Figure 2:**
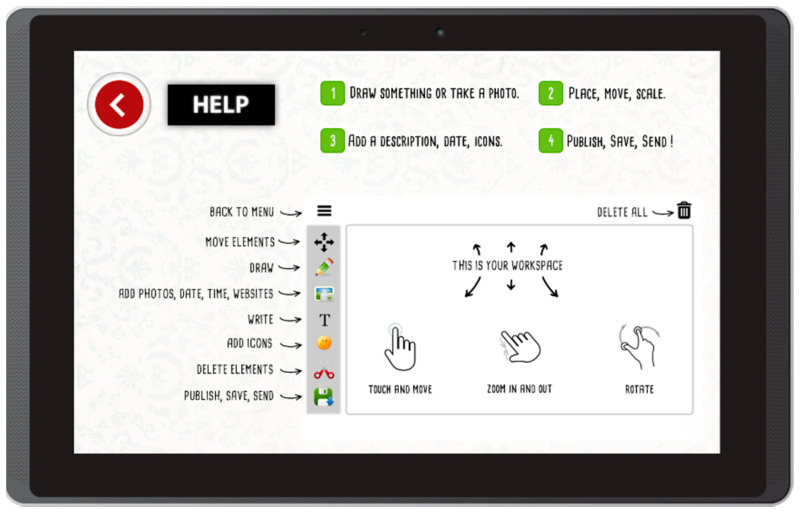
Help screen in Aphasia Create (screenshot from an Android tablet).

The Help button leads to a screen with a description of all available interactions (Figure 2).

**Figure 3:**
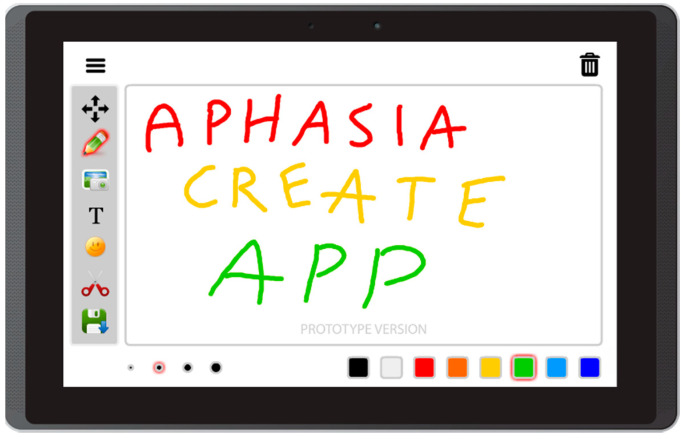
Free drawing and taking quick notes tools in Aphasia Create (screenshot from an Android tablet).

**Figure 4:**
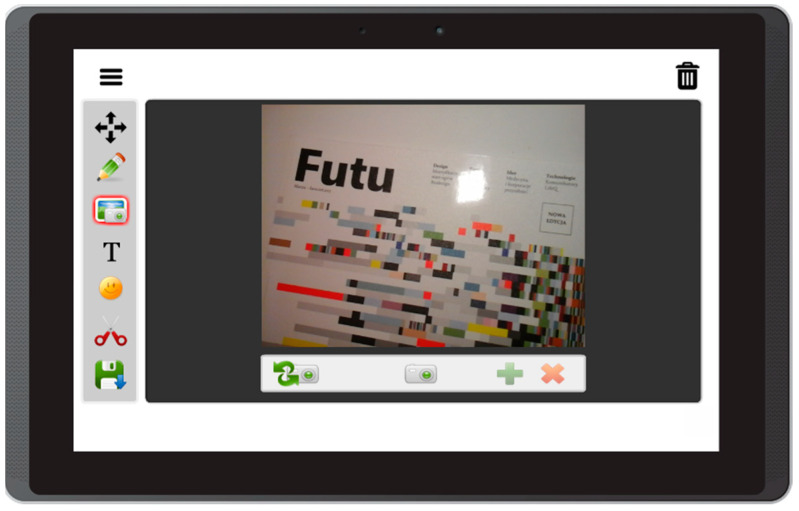
Taking photos with the front and back camera in Aphasia Create (screenshot from an Android tablet).

**Figure 5:**
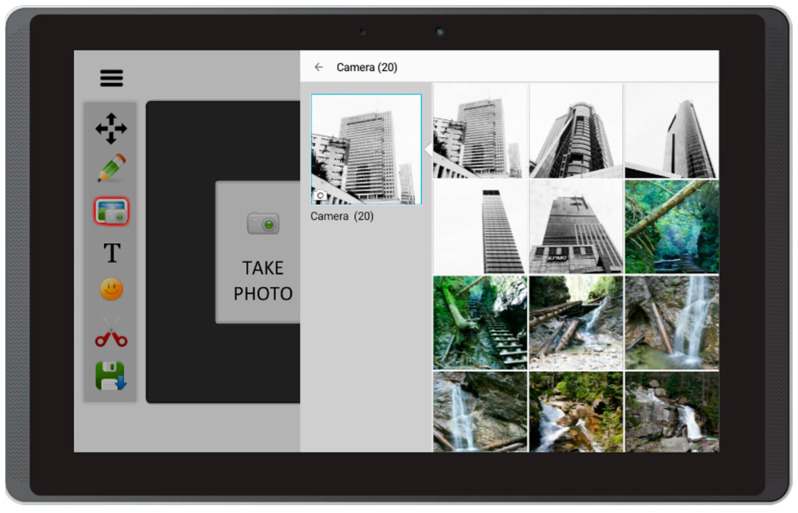
Accessing camera memory for loading and saving images in Aphasia Create (screenshot from an Android tablet).

**Figure 6:**
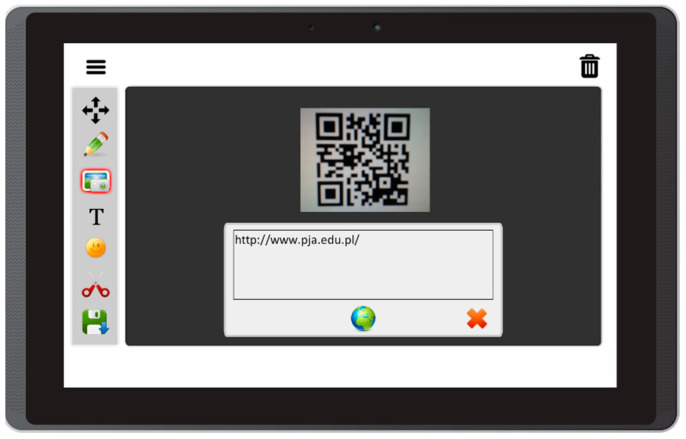
Reading QR codes for opening websites in Aphasia Create (screenshot from an Android tablet).

**Figure 7:**
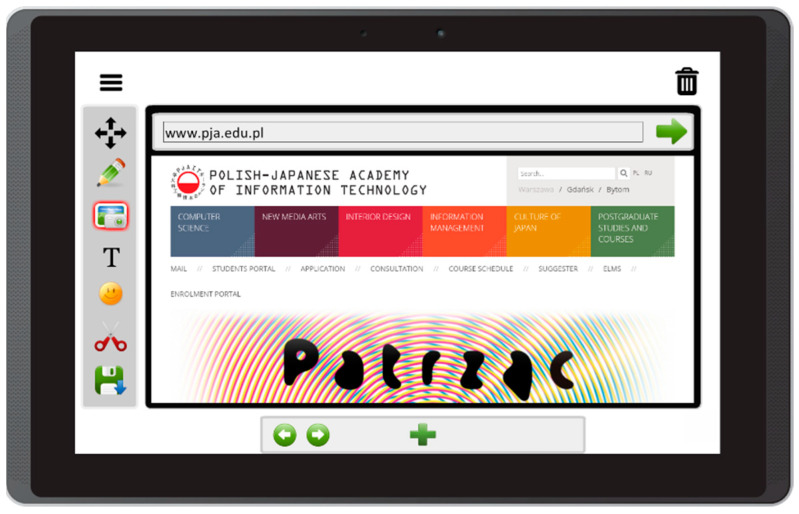
Accessing websites in a web browser in Aphasia Create (screenshot from an Android tablet).

**Figure 8:**
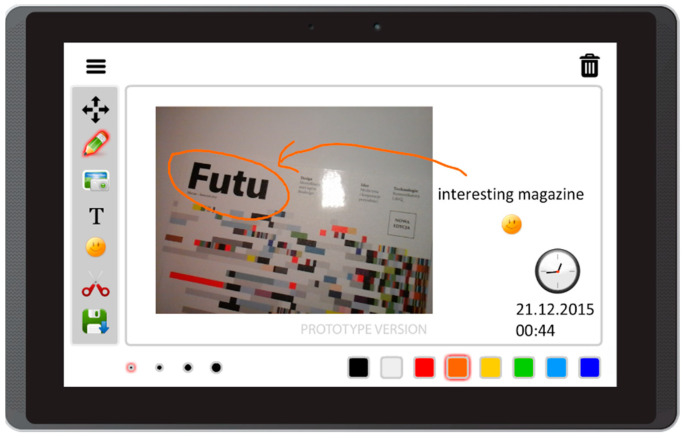
Adding text, emoticons, and time stamps in Aphasia Create (screenshot from an Android tablet).

**Figure 9:**
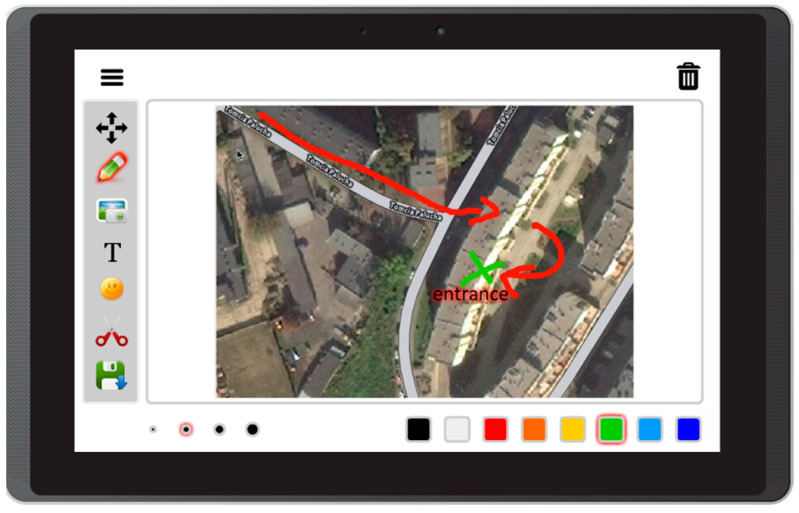
Adding maps and notes in Aphasia Create (screenshot from an Android tablet).

**Figure 10:**
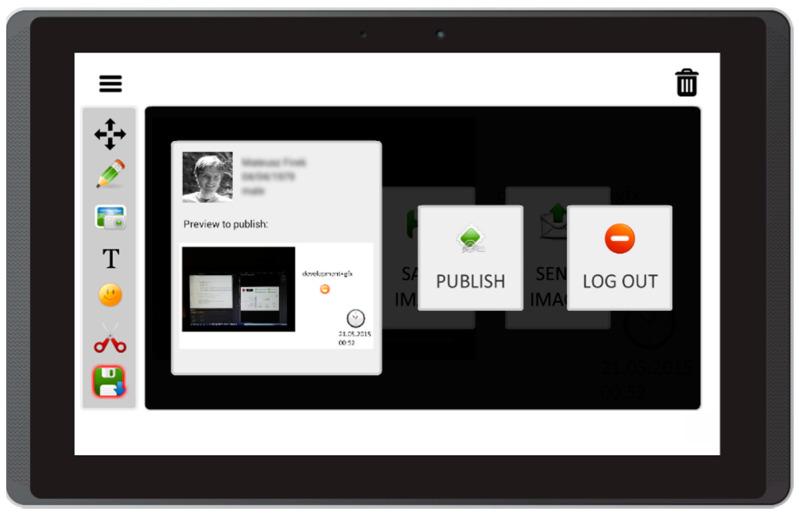
Publishing a canvas to Facebook with an easy-to-use interface and sending emails in Aphasia Create (screenshot from an Android tablet).

Based on the literature review concerning the usage of mobile devices for storytelling, and on interviews with caregivers of people with aphasia, this prototype app offers the following features:

- Free drawing and taking quick notes—people often need to communicate something quickly, and this can be implemented in the form of sketches or words written on the screen (Figure 3);
- Taking photos with front and back camera—when the user is in an interesting spot, they can take a photo and send it or share it immediately. Another option is to save it for later use in the communication process (Figure 4);
- Accessing camera memory for loading and saving images—this feature allows the creation of visual messages, constructed based on previously taken photos (Figure 5). The user can save created images to the camera memory for future reuse or sharing;
- Reading QR codes for opening websites in a mobile browser—people who have problems with reading/writing can use this feature to open websites (Figure 6);
- Accessing websites in a web browser—since the app is integrated with a web browser, the user does not have to open an external application. This also enables taking screenshots of interesting webpages and using them to build a visual message (Figure 7);
- Adding text, emoticons, and time stamps—the user can add text. All operating system (OS) features are available, including spell checking and speech synthesis. Patients who have retained their writing ability can enter text using the keyboard and put it on the canvas. The user can also add emoticons, commonly used for expressing emotions about their experiences. Additionally, since some patients have problems with remembering events and places, adding time stamps (current date and time) can help them to organize events on the timeline and recall them later (Figure 8);
- Accessing maps with geolocation—the application is integrated with OS maps, so the users may quickly check their position, and can also take a screenshot to put it on the canvas (Figure 9);
- Publishing the canvas to Facebook with an easy-to-use interface, and sending emails—the users can form simple stories by using these tools, and can share their experiences of everyday life with other people using social services, or send emails containing graphical representation of the story (Figure 10).

When designing the application, the guidelines listed below were followed:

- The position of the interface and controls should be on the left side, due to frequent paralysis of the right hand;
- The application should be operable with one hand;
- Interface elements should always be available and easy to understand;
- The control elements and icons should be large and meaningful.

### 2.2. Aphastory Application for Google Glass

Although the technology use of persons with aphasia can be low prior to therapy [26], there is still the potential to apply wearable devices to help patients with aphasia. Devices in the form of glasses can be especially useful, as these are hands-free devices, and the images seen by the person wearing the device can be transmitted to others. Examples of such smart glasses include Epson Moverio [27], Microsoft HoloLens [28], and Google Glass [29]. Google has authorized numerous partners to deliver enterprise solutions for Google Glass; providers include medical and paramedicine companies, such as Augmedix [30] and swyMed [31]. Additionally, Stanford Medicine has applied Google Glass in The Autism Glass Project for autistic children [32]. Since Google Glass is applied in medicine and therapy [33], we also decided to use this device, based on our prior experience in application prototyping for Google Glass [34].

Aphastory is a storytelling application for Google Glass, designed for persons with aphasia; it was created using the Android Studio development environment. Our main goal was to test to what extent wearable devices such as Google Glass can support persons with aphasia in sharing their daily experiences and activities, and in improving the quality of their lives. Because of the considerable communication problems of persons with aphasia, we decided to acquire the knowledge needed from the neuropsychologists working with these patients, as well as from the literature [14,16,17,26,33].

The application’s concept is based on observations and experience gained from the development of the Aphasia Create app for tablets. Unfortunately, the Google Glass device does not offer such extensive functionalities as the tablet device. However, the Aphastory app preserves the most important functionalities, so the user can:

- Take pictures and record videos using a built-in camera;
- Create stories composed of photos and videos as sequences that follow one another;
- Annotate individual photos or videos with short captions, icons, and geolocation;
- Save the created story in the internal memory of the device;
- Send the created story to an email address.

Interaction with the application is performed through tap and swipe gestures on the device’s touchpad. Naturally, voice interaction was not implemented, since the users with aphasia have difficulties with vocal communication.

The stories are created in the form of a show of consecutive pictures and/or movies, which can be accompanied with predefined phrases and icons, helping in narration. Geotagging is also available for each story element. Such a package can be saved or sent via email.

**Figure 11:**
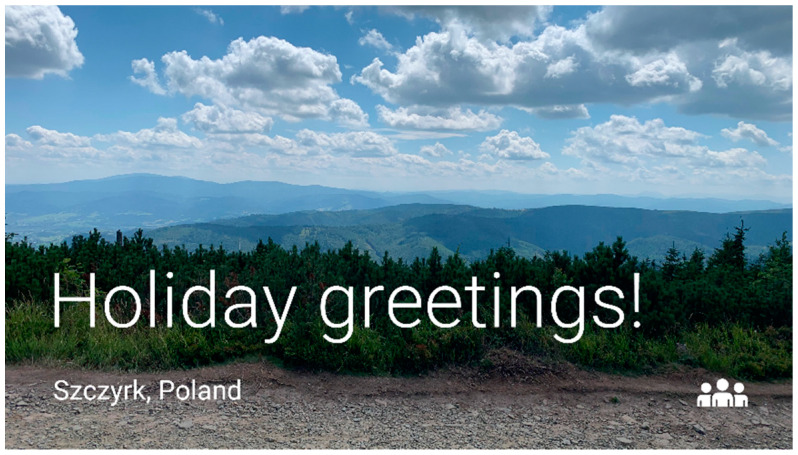
Story fragment consisting of a picture, caption, icon, and geolocation in Aphastory (screenshot from a Google Glass device).

Figure 11 presents a story fragment, consisting of a picture, caption, icon, and geolocation; the application is prepared for Polish users, so the interface and text elements are all in Polish. The video presenting the use of Aphastory is also available for interested readers (https://bit.ly/3HgmHdj, accessed on 16 December 2021).

### 2.3. Evaluation Methods of the Aphasia Create and Aphastory Apps

**Figure 12:**
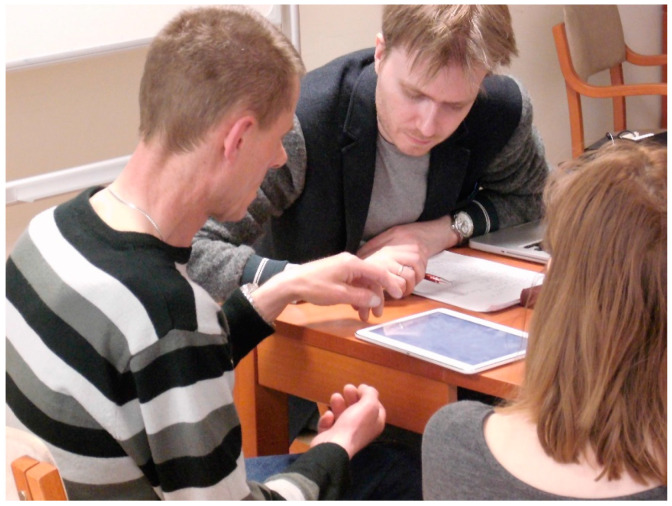
User tests with people with aphasia in Poznań.

Before the applications were tested for usability, the Aphasia Create app was pre-evaluated by carers and target users; it was introduced to 10 persons with aphasia to get their first impressions, with the help of an aphasia association based in Poznań, Poland [35]. Users were familiarized with the application and encouraged to ask questions while performing simple tasks. The purpose of this preliminary study was to find out what information the users were missing and what feelings their first contact with the application on the tablet raised. It was also important to check how to formulate tasks and questions in conversation with people with aphasia during future usability tests. In particular, this influenced the form of asking questions in the final phase of the satisfaction survey. People with aphasia have difficulty understanding sentences of complex structure. Therefore, it is best to get feedback by making a simple statement and asking to what extent the person agrees or disagrees with it. Figure 12 shows a meeting with one of the respondents and a conversation about the application.

The elderly, due to their lack of familiarity with mobile devices, had problems in the initial phase of touchscreen operations, but after several attempts they quickly learned how to use the application. Persons with more technical experience did not have problems with using the application. Due to the ease of access to the interface elements, it was better to use tablets with a display size of approximately 10 inches or more. For most testers, this was their first contact with this type of application, and they appreciated the combination of these features without having to use several programs to achieve the goal as they did before.

After the pre-evaluation, we prepared and conducted usability tests. These tests were carried out on 6 people. According to Jakob Nielsen’s research [36], even 5 testers should be sufficient to find most of the critical errors in the application. Since persons with aphasia suffer from movement and sensory limitations, we tested both apps as tools for such persons. We observed how the users reacted, and how they progressed when using the application. As a result, we obtained comments from the users that indicated how the applications could be improved.

### 2.4. Participants

In order to obtain reliable results, we selected patients with aphasia treated to the extent allowing communication with the researcher. Therefore, we performed usability tests with 6 persons with aphasia, aged 30–66 years—5 women, and 1 man (Table 1). These patients represented various education levels, aphasia types, and experience in using mobile devices. The tests were performed at the Polish–Japanese Academy of Information Technology in Warsaw, Poland.

All of the investigated patients underwent treatment for aphasia; this treatment can last many years (and can be lifelong). Our testers had already finished their treatment, or were still undergoing it. Additionally, they were no longer hospitalized, and they were trying their best to lead normal lives, regardless of the difficulties and side effects of aphasia.

### 2.5. Usability Tests of the Aphasia Create App

Due to the low popularity of the Google Glass device (especially among the elderly), and in order to present the idea of storytelling, participants were asked to familiarize themselves first with the Aphasia Create tablet app described above.

For this purpose, each of the subjects had to perform several simple tasks:

1. Start the application;
2. Understand functions on the tools panel;
3. Draw simple shapes;
4. Change the drawing color;
5. Change the brush thickness;
6. Select objects on the scene;
7. Delete objects from the scene;
8. Take a picture using the built-in camera;
9. Add a picture to the scene;
10. Resize objects using gestures;
11. Load photos from the gallery;
12. Open a particular website;
13. Add a screenshot of the website to the scene;
14. Write text and add it to the scene;
15. Add a time and date to the scene;
16. Add an emoticon to the scene;
17. Save/send the created scene.

Each session was ~30 min long, and was attended by the tester (person with aphasia), the researcher, and the neuropsychologist. The following scale was adopted to assess the performance:

- Failure (0)—the participant failed to complete the task (even a fragment);
- Partially completed (1)—the participant completed part of the task with the help of the researcher;
- Success (2)—the participant completed the task entirely unassisted.

### 2.6. Usability Tests of the Aphastory App

After a short break, the subjects were asked to perform tasks with the Google Glass device. As in the previous case, the session was ~30–45 min long, and was attended by the tester (person with aphasia), the researcher, and the neuropsychologist.

To start with, the tester was familiarized with the device and how to control it. Initially we planned to have 10 tasks performed, after the tester was familiarized with controlling the device. However, after the first test we found that 10 tasks were far too many, and that the tests would take too long. Therefore, we limited the test to 5 tasks, testing only the most important functionalities of the application. The difficulty level of these tasks increased gradually from task to task. Finally, the summarizing task was performed, in order to check whether the tester understood the main functionalities of the application well. All testers completed at least 3 tasks, depending on their advancement level. The following tasks were given to the testers:

- Create a new story, consisting of one picture taken, and save it;
- Add a movie (no more than 20 s) to the existing story. Save the story;
- Create a new, more advanced story, containing a picture from the gallery. Assign a caption, location, and icon;
- Save the story created recently in the menu “My stories”. Display the selected story;
- Remove the selected story, using the menu in “My stories”;
- Send the created story to any contact.

### 2.7. Post-Test Survey

After the test, the users filled in a questionnaire regarding the ease of working with both applications. Initially, we wanted to use the System Usability Scale (SUS) [37]. The original SUS questionnaire has 10 questions, with mixed positive and negative statements, and a 5-point Likert scale is used for answers. After consultations with a psychologist, we were instructed that the communication with these patients requires simplified language, short sentences, and slow speaking. Therefore, the questions in the scale had to be limited and simplified, as they were designed for disabled persons. Participants answered the following questions:

1. The application may be useful for people with aphasia;
2. Devices of this type have great potential for people with aphasia;
3. The application was easy to use;
4. The operation of the user interface was understandable;
5. The story-making features were adequate.

Answers were given on a 5-point Likert scale: strongly disagree, disagree, neutral, agree, or strongly agree. At the end of the test, open-ended questions were asked about what the participants liked or did not like about the presented application, and what functions could be added to it.

## 3. Results

### 3.1. Aphasia Create Usability Test Results

**Figure 13:**
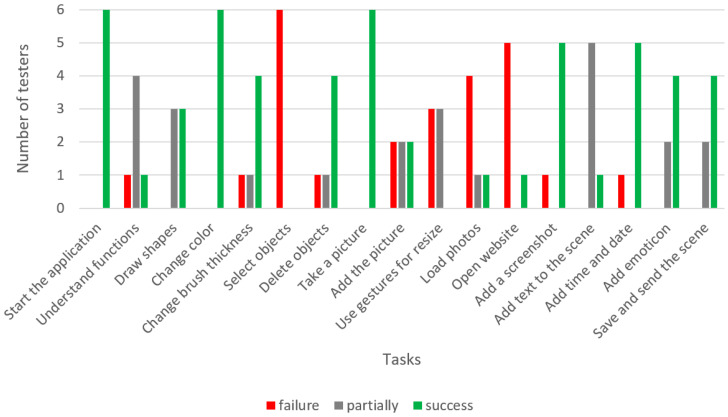
Results of the usability test of the Aphasia Create app.

There were visible deficiencies in the ability to use tablet touchscreens during this study. This problem definitely disturbed the understanding of the application’s operation, because the operation of the device itself was troublesome. For example, several participants had a problem with touch and typical gestures used in tablets (e.g., people tried to press the screen buttons for a long time, as if they were physical buttons, and were not familiar with commonly used gestures to resize screen objects). Similarly, some testers did not associate the meaning of standard icons, e.g., icons with arrows for selecting and moving objects, floppy disk icons for saving documents, icons with the letter “T” for activating text tools, etc. The usability test results of the Aphasia Create application are presented in Table 2 and Figure 13.

The least problematic tasks were:

- Starting the application;
- Changing the drawing color;
- Taking a photo;
- Adding a screenshot to the scene;
- Adding time and date;
- Adding emoticons to the scene;
- Saving and sending the scene.

Tasks that most users managed to perform with the help of a researcher were:

- Drawing simple shapes;
- Changing the brush thickness;
- Deleting objects;
- Writing text and adding it to the scene;
- Understanding the function of the application;
- Adding a photo to the scene.

The most problematic tasks were:

- Resizing objects using gestures;
- Loading photos from the gallery;
- Opening a website;
- Selecting objects.

During the study, several technical and design errors were noticed:

- In some cases, the device did not respond quickly enough to touch, and even crashed;
- In the drawing tool, the choice of brush colors and thickness is located near the bottom edge of the screen, and participants sometimes accidentally activated the built-in tablet menu;
- Incorrect implementation of object selection—participants had problems with the precise selection of objects, especially if there were a lot of them on the screen;
- Selecting objects is not intuitive—it was difficult to recognize whether an object was selected, and it was sometimes moved accidentally;
- Some functions are not intuitively grouped, e.g., launching a website is in the photo importing section, and sharing the screen content is in the screen saving section.

**Figure 14:**
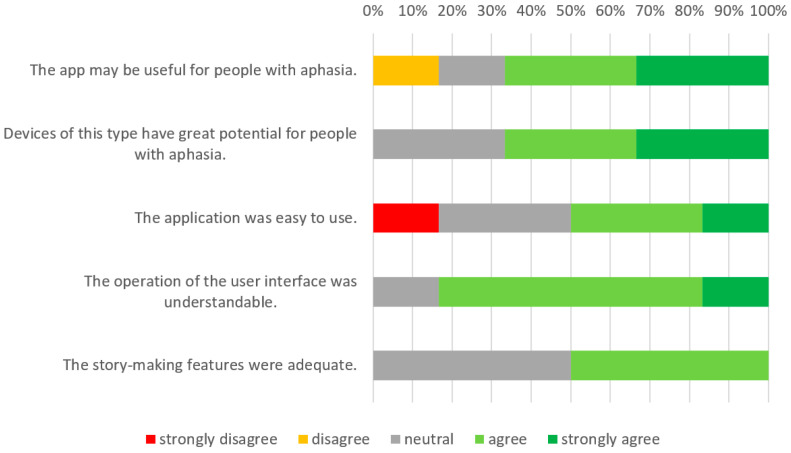
Results of the post-test survey for the Aphasia Create app.

Results of the post-test survey are presented in Figure 14. The survey confirmed a positive attitude towards the application. The participants in particular liked taking photos and adding emoticons, as well as describing the created compositions. They noted, however, that the interest in tablets among the elderly is low, and that the support of more experienced people would be necessary at the beginning.

### 3.2. Aphastory Usability Test Results

The first contact with the Google Glass device was stressful for the users, but over time the control of the device became easier. At the beginning, it was problematic to understand how to navigate the application menu—especially how to navigate through the various menu levels and within a given category. After familiarizing the users with controlling the application, it was easy for them to find the options that they were looking for. Commands were relatively easy to complete, and the testers appreciated the ease of taking pictures and videos. The options available for creating stories were sufficient, and the stories were well organized and easy to view.

However, the users experienced problems when using the touchpad. The persons with sensory disorders were not able to identify the touchpad, they could not see what they were touching, and they had difficulties in navigating within the application. The device turned out to be too sensitive, or not sensitive enough to register the touch, or did not convey the touch signal fast enough.

Another problem was the position of the touchpad (on the right-hand side). When the left hemisphere is damaged, it results in paresis of the right hand. The touchpad of Google Glass is located on the right-hand side, and there is no option to change this position. Therefore, the testers with right-hand paresis had to control the device with their left hand. Still, these users were able to learn how to control the device. Google Glass was designed for right-handed users, which is the drawback of this device.

**Figure 15:**
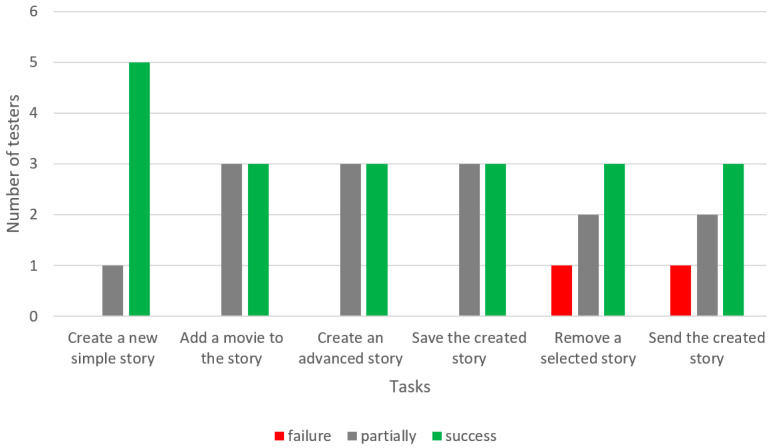
Results of the usability test of the Aphastory app.

The users also reported problems with feedback within the application. Specifically, they had doubts as to whether an action was performed correctly in the application. They reported the need to display messages confirming whether their commands were performed (e.g., the picture or video or story was saved). The usability test results of the Aphastory application are presented in Table 3 and Figure 15.

The least problematic task was:

- Creating a new simple story.

Tasks that most users managed to perform with the help of a researcher were:

- Adding a movie;
- Creating a new, more advanced story;
- Saving the created story.

The most problematic tasks were:

- Removing a selected story;
- Sending the created story to any contact.

**Figure 16:**
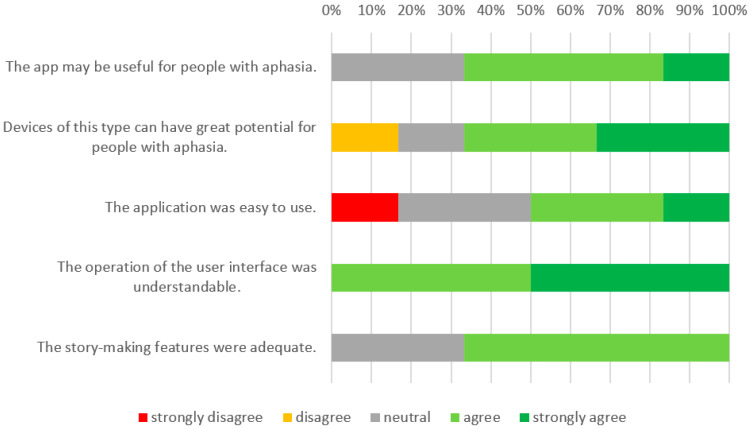
Results of the post-survey test of the Aphastory app.

The results of the post-test survey are presented in Figure 16. The survey, as in the case of the study for the tablet application, confirmed the positive attitude towards the app. Participants particularly enjoyed creating stories using photos and videos, and being able to describe them. However, they noted that the Google Glass device is a very unusual device, and requires getting used to wearing and handling it.

## 4. Discussion

When designing applications for people with expressive aphasia (Broca’s aphasia), we have noticed some important remarks that may be of use to future designers.

### 4.1. Participatory Design Is Key to Design for People with Aphasia

Thanks to conversations with a neuropsychologist and caregivers of aphasics, it was much easier to understand their everyday lives and needs. Talking to people with aphasia face-to-face must be very simple, without the use of advanced sentence structure. Moreover, aphasics differ from one another in their disorders, and it is helpful to discuss their specific needs together with caregivers.

### 4.2. A Simplification of the User Interface and Its Structure Is Required

Unfortunately, aphasia most often affects the elderly, who may be less tech-savvy. This is why young users found it easier to use both applications, as they had used portable devices in their everyday lives. Therefore, the user interface and interaction with apps should be as simple as possible, and should present the most important functions in an understandable way. Too many elements will be distracting and discouraging. The icons used should ideally fit the metaphors of the tasks that can be performed. The number of steps to be performed during the task and the screens that present them should be kept to a minimum.

### 4.3. The Paralysis of the Right-Hand Side of the Body Must Be Taken into Account When Designing the Interface

Due to impairments of the right-hand side of their body (and sometimes also right-hand side vision), it is suggested to design the control elements at the left-hand side of the interface. The device should be capable of being operated with the left hand only. Handling touchscreens can also be a challenge, so interface objects should be large enough, separated by empty space, and not placed close to the edges of the screen. It is not recommended to use gestures. The tests also indicated that it was better to use tablets with a display size of approximately 10 inches or more, due to the ease of access to the interface elements.

### 4.4. Storytelling Functions Engaged People with Aphasia, and Their Use Was Interesting for Them

The respondents expressed the greatest interest in taking photos, drawing, and adding times, dates, and emoticons to the scene. This gave them a lot of fun, and they willingly repeated these tasks without even being asked to do so. The patients were inventive in creating their own stories on mood boards, and they enjoyed describing them with drawings and icons. They also indicated these tasks as being the most interesting in the final evaluation questionnaire.

### 4.5. Popular Storytelling Mobile Apps Do Not Meet the Requirements of People with Aphasia

Typical applications for aphasia are designed to be used as rehabilitation tools aimed at regaining language skills, whereas our apps are designed to help in communication through storytelling. Our conversations with caregivers and patients showed that the mainstream storytelling applications are not tailored to the specific needs of older people with aphasia, as these apps lack important functionalities, and are too complicated for such users. Our research also results from the literature on the subject. The disadvantages of the aforementioned applications—such as Day One, Five Minute Journal App, etc.—are as follows: multiscreen and multilayer interfaces; no possibility to create mood boards with various objects, such as photos, icons, text, clocks, and dates; no possibility to draw on photos; no icons or emoticons to label emotions; a large amount of small text in the user interface; very closely spaced icons close to the edges; a difficult-to-understand process of creating entries, with too many elements at once; etc. Considering applications on Google Glass, such as Memoirs or Glossaic, they do not offer the possibility to create stories from sequences of photos and videos, and they do not have the option of describing photos with icons or text.

### 4.6. The Use of Smart Glass Devices Can Be Valuable, but It Does Have Its Limitations

The Aphastory app for Google Glass could be helpful for the patients in maintaining their social bonds and, thus, improving their quality of life; however, it has several limitations. Specifically, as mentioned above, many patients with aphasia suffer from hemiparesis or apraxia involving their dominant hand, which makes it difficult or sometimes impossible to efficiently control the touchpad located on the right-hand side of the device. Moreover, aphasia is an impairment of the whole language system, not merely a speech disability; therefore, reading and understanding commands, as well as choosing captions for photos, may be beyond the reach of some patients. 

The virtue of Google Glass is the comfort of using it, as it is light and is not handheld. This device is also an interesting solution for users who do not have a computer. Google Glass enables staying in touch with friends, with minimal effort applied. The application could be easily used by active persons who walk or travel a lot, and would like to share their experience with their families. Therefore, Aphastory is a promising application for some patients—preferably young, without general cognitive decline, and with mild aphasia.

### 4.7. Usability Testing Methods

Testing an application’s usability with people with aphasia requires patience and empathy. The performed tests had to be of a less formal nature due to the patients’ disease, which made it difficult to communicate with them during the tests. Before performing the tasks, we discussed the operation of the devices and the purpose of the study. Tasks to be performed should be short and understandable. When performing the task, it is worth making sure that the aphasic knows what they are doing and remembers the purpose of the task. It is useful to present the real context of the performed task in order to better explain its purpose. The experiment is more like going through tasks together with the person than a typical timed usability test. The form of the final evaluation is also very important. In our case, we used selected SUS-inspired questions that were simplified after consulting with a neuropsychologist.

## 5. Conclusions

We started our work with the analysis of the available storytelling software solutions, aiming at obtaining applications designed for persons with aphasia in order to improve communication by storytelling methods. During the research, we drew particular attention to the problems and needs of persons with aphasia—especially the elderly. We also took into account the typical functionalities of mobile and wearable devices, and how they could be used for building robust applications with an easy-to-use interface. Since no storytelling apps for persons with aphasia were available, we designed and implemented two prototypes of simple storytelling applications that help to share the experiences of everyday life with other people, using social services. The first application—Aphasia Create—was designed for mobile devices such as tablets; it has been recognized by experts, and won the Enactus Poland National Competition in 2015 [38]. The second app—Aphastory—was designed for the Google Glass device [39,40].

Our designed applications confirmed that (1) storytelling is an unmet need of individuals with aphasia, and current mainstream solutions do not support them in this regard; (2) the first reactions to the presented projects were very positive, and people with aphasia would like to use our applications to improve the quality of their lives through storytelling methods; (3) the most willingly used functions were taking pictures and videos and then describing them with drawings, emoticons, etc.; (4) thanks to the appropriate selection of limited functionalities, screen layout, icon design, and simple workflow of interactions, mobile applications targeted at people with aphasia can better fulfil their needs; and (5) using a tablet application would be more affordable than the smart glass version (the Google Glass device is rather suitable for young and active people who can operate with their right hand).

In future works, we would like to improve the means of creating and sharing stories, in order to prepare effective and intuitive methods for reconstructing everyday events. We believe that long-term follow-up is needed in order to better visualize what functionalities are most needed by people with aphasia. We anticipate that advances in tablet or smart glass technologies will contribute to even greater popularity of these devices, even among the elderly. This may result in the possibility of reaching potential recipients more easily, and offering them new solutions even better suited to their needs.

## Figures and Tables

**Table 1:** Usability test participants.

ID	Sex	Age	Education	Dominant Hand	Aphasia	Time Since Stroke (Years)	Experience with Mobile Devices (Except Mobile Phones)	Symptoms
1	F	49	Incomplete higher	Right	Sensory	9	No	Sensory problems and issues with information analysis
2	F	48	Vocational	Right	Sensory	<1	No	Problems with understanding and information analysis
3	F	60	Incomplete higher	Right	Sensory	14	No	Sensory disorders
4	M	65	Higher	Right	Sensory	6	Little	Right hand paresis; problems with speech and information analysis
5	F	30	Incomplete higher	Before aphasia, right; now left	Mixed	5	No	Right hand paresis; problems with speech and information analysis
6	F	66	Secondary	Right	Sensory	12	No	Sensory disorders

**Table 2:** Results of the usability test of the Aphasia Create application.

Task	User 1	User 2	User 3	User 4	User 5	User 6
Start the application	Success	Success	Success	Success	Success	Success
Understand functions	Partially	Partially	Partially	Success	Partially	Failure
Draw shapes	Partially	Partially	Success	Partially	Success	Success
Change color	Success	Success	Success	Success	Success	Success
Change brush thickness	Success	Success	Success	Failure	Success	Partially
Select objects	Failure	Failure	Failure	Failure	Failure	Failure
Delete objects	Failure	Partially	Success	Success	Success	Success
Take a picture	Success	Success	Success	Success	Success	Success
Add the picture	Failure	Success	Partially	Partially	Failure	Success
Use gestures for resize	Failure	Partially	Partially	Partially	Failure	Failure
Load photos	Failure	Failure	Success	Failure	Failure	Partially
Open website	Success	Failure	Failure	Failure	Failure	Failure
Add a screenshot	Success	Success	Success	Success	Failure	Success
Add text to the scene	Partially	Partially	Success	Partially	Partially	Partially
Add time and date	Success	Success	Success	Success	Failure	Success
Add emoticon	Success	Success	Success	Partially	Partially	Success
Save and send the scene	Success	Partially	Success	Success	Success	Partially

**Table 3:** Results of the usability test of the Aphastory application.

Task	User 1	User 2	User 3	User 4	User 5	User 6
Create a new simple story	Success	Success	Partially	Success	Success	Success
Add a movie to the story	Success	Partially	Partially	Success	Partially	Success
Create an advanced story	Success	Partially	Partially	Success	Partially	Success
Save the created story	Success	Partially	Partially	Success	Success	Partially
Remove a selected story	Partially	Partially	Failure	Success	Success	Success
Send the created story	Partially	Failure	Partially	Success	Success	Success

## Data Availability

Not applicable.
